# Case report: Basal cell carcinoma arising within the nevus sebaceous: report of 4 cases and literature review

**DOI:** 10.3389/fonc.2024.1478747

**Published:** 2025-01-07

**Authors:** Tianbing Lei, Yonghong Lu, Yanyan Feng, Yao Ni

**Affiliations:** Department of Dermatovenereology, Chengdu Second People’s Hospital, Chengdu, Sichuan, China

**Keywords:** nevus sebaceous, basal cell carcinoma, malignant degeneration, gene, ultraviolet

## Abstract

Nevus sebaceous (NS) is a congenital hamartoma characterized by the presence of skin structures, including the epidermis, sebaceous glands, and hair follicles. NS predominantly occurs on the scalp and has the potential to give rise to secondary tumors, with a small proportion being malignant; the most frequently observed malignant tumor associated with NS is basal cell carcinoma. In this report, we retrospectively present four cases of sebaceous nevus on the scalp complicated by basal cell carcinoma. The pathological diagnoses were unequivocal in all four patients, who exhibited typical clinical manifestations, with all cases localized to the left parietotemporal region of the scalp. The etiology of NS may be linked to genetic mutations or ultraviolet exposure. Treatment options vary, with surgical resection likely being the most common method of eradication.

## Introduction

Nevus sebaceous (NS) is a common congenital hamartoma of the skin, primarily composed of sebaceous glands. It typically occurs on the scalp, face, and neck, with a prevalence ranging from 0.05% to 1% (1–3). NS often presents at or shortly after birth, predominantly on the scalp, followed by the face, with fewer cases affecting the neck and trunk. In some instances, multiple plaques or nodules may be observed, appearing as clearly demarcated orange-yellow patches devoid of hair growth. While most NS are benign skin tumors at the time of diagnosis, a small percentage may progress to malignant lesions. The most frequently associated malignancy is basal cell carcinoma (BCC), followed by squamous cell carcinoma (2). The reported incidence of malignant transformation of NS into BCC is 0.8% (1). Although BCC constitutes a minor proportion of secondary tumors arising from NS, it represents the highest incidence among NS-related malignancies, underscoring the importance of early diagnosis. Given its invasive nature and the importance of early detection and management, heightened clinical attention and vigilance are warranted. In this report, we present four cases of left scalp NS with BCC.

Among the four cases reported, all patients exhibited left parietal-temporal scalp plaques and grayish-black masses. The diagnosis of head NS complicated by BCC was confirmed through systematic specialty examination and histopathological analysis. This study received approval from the Ethics Committee of Chengdu Second People’s Hospital (KY-PJ2024061). Written informed consents were obtained from the patients for the publication of this case reports.

## Case report

### Case 1

A 46-year-old female patient presented to a dermatology clinic with a flaky patch on the left parietal temporal scalp that developed over 30 years ago. The lesion has been accompanied by occasional itching, which has not been treated. In the last two years, the patient has noticed a flaky black covering in the center of the lesion, without any identifiable cause. There have been no reports of local itching, rupture, erosion, or seepage. The patient has been previously healthy and has no family history of similar conditions.

**Figure 1:**
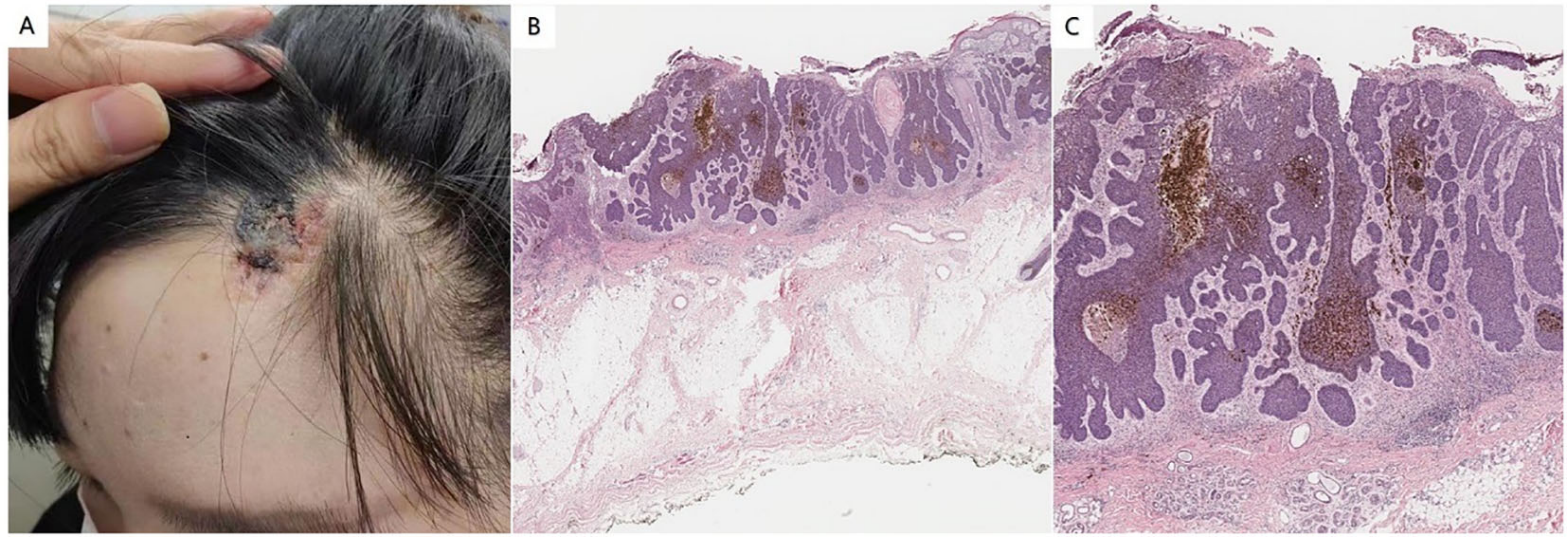
**(A)** Soft yellowish patches were observed on the left scalp. A flaky black nodule was observed in the lesion. **(B)** Histopathology showed epidermal erosion and necrosis, and basal-like cell tumor masses were seen in the superficial middle layer of the dermis, some of which were connected to the epidermis (hematoxylin and eosin staining; magnification:×100). **(C)** The tumor cells are small, the nuclei are deeply stained, round or oval, the shape is uniform, and the cytoplasm is less. There was no bridge between the tumor cells, the cells around the tumor were arranged in a palisade, and some tumor masses had pigmentation (hematoxylin and eosin staining; magnification:×200).

The physical examination confirmed the absence of abnormalities in the system. The dermatological assessment revealed a soft, yellowish patch measuring approximately 3.2 cm × 2.7 cm on the left scalp, accompanied by a flaky black nodule measuring 2.0 cm × 2.5 cm within the lesion (**Figure 1A**). Histopathological analysis indicated epidermal erosion and necrosis, with basal-like cell tumor masses observed in the superficial middle layer of the dermis; some of these masses were connected to the epidermis. The tumor cells were small, with deeply stained, round or oval nuclei that exhibited uniformity, and the cytoplasm was minimal. There were no connections between the tumor cells, while the surrounding cells were arranged in a palisade formation, and some tumor masses displayed pigmentation (**Figures 1B, C**). These findings are consistent with a diagnosis of NS complicated by BCC. The classification of this BCC was nodular.

The treatment involved an extended surgical resection, during which a frozen section was sent for intraoperative analysis. The incisal margin and base were thoroughly cleaned, and the flap was subsequently repaired. The patients were monitored for one year post-discharge, during which time healing was satisfactory and no recurrences were observed.

### Case 2

A 49-year-old woman presented to a dermatology clinic with a nodule on the left side of her scalp. She reported having a plaque on this area since childhood, with no associated symptoms and no systemic treatment. Over the past year, several round patches with gray and black edges developed, raised in the center of the lesions without any obvious cause. These patches gradually enlarged, forming black nodules, which were accompanied by mild pain, itching, and some bloody exudation following scratching. The affected area had been treated multiple times with cryotherapy, but the results were unsatisfactory. The patient was in good general health and had no family history of similar conditions.

**Figure 2:**
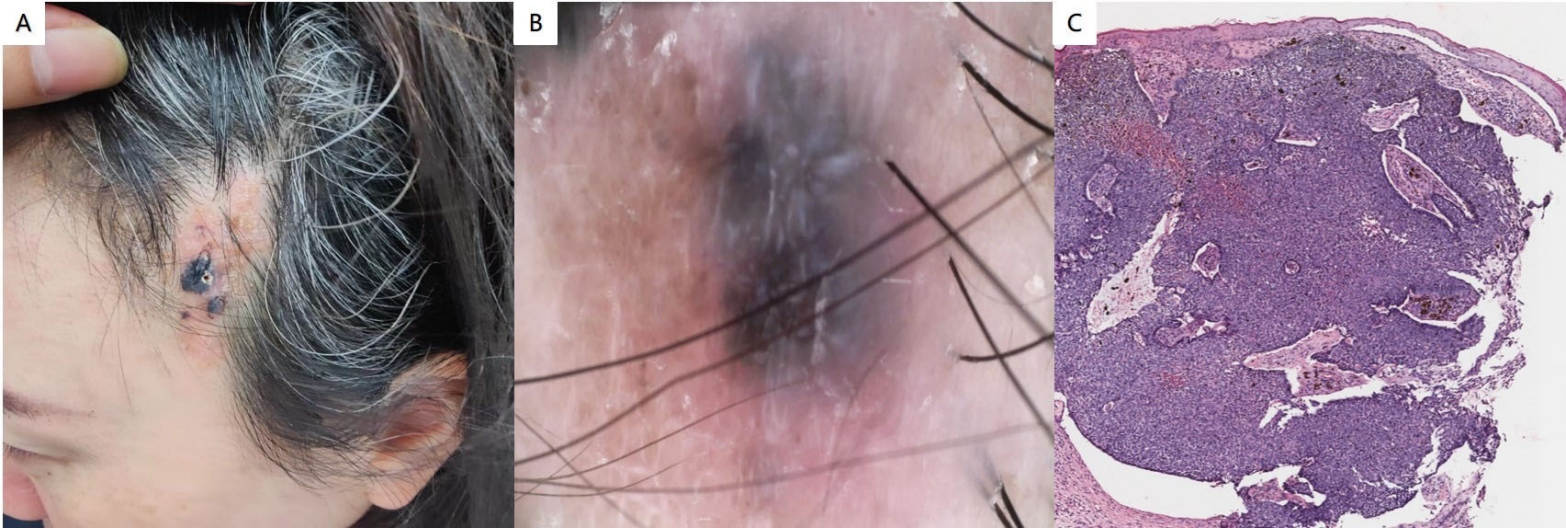
**(A)** Soft yellowish patches were observed on the left scalp, accompanied by two black nodules within the lesions, which exhibited clear boundaries and a raised surface. **(B)** The dermoscopy revealed a large bluish-gray oval nest set against a pink background, along with shallow ulcers and a few localized white scales. **(C)** Histopathological findings indicated that the epidermal mutation was flat, with tumor cells displaying clear boundaries located in the dermis, most of which were connected to the epidermis. The morphology of the tumor cells resembled that of basal cells, and the surrounding cells were arranged in a palisade pattern. Additionally, artificial contraction spaces were noted, along with evidence of bleeding and pigmentation within the tumor (hematoxylin and eosin staining; magnification:×100).

The system check is normal. The dermatological examination revealed soft, yellowish patches measuring approximately 3.2 cm × 5.2 cm on the left scalp. Two black nodules, measuring 1.1 cm × 1.2 cm and 0.5 cm × 0.5 cm, were observed within the lesions, characterized by clear boundaries and a raised skin surface (**Figure 2A**). Dermoscopy revealed a large bluish-gray oval nest on a pink background, shallow ulcers, and a few white scales, along with multiple bluish-gray bodies. Notably, there were no dendritic telangiectasia or pigmented webs present (**Figure 2B**). Histopathological findings indicated that the epidermal mutation was flat, with tumor cells exhibiting clear boundaries visible in the dermis, most of which were connected to the epidermis. The morphology of the tumor cells resembled that of basal cells, and the surrounding cells were arranged in a palisade pattern. Additionally, artificial contraction spaces were noted, along with evidence of bleeding and pigmentation within the tumor (**Figure 2C**). This description is consistent with a diagnosis of NS complicated by BCC. The tumor was classified as nodular.

The left scalp lesion was treated with an extended excision, and a frozen section was obtained during the procedure. Following confirmation that the incision margins and base were clear, the flap was repaired. The sutures at the surgical site healed well, and no recurrence was observed three months post-operation.

### Case 3

A 46-year-old female patient presented to the dermatology department with a lesion located on the left parietotemporal area of the scalp. At the age of 4, the patient developed a red papule in the left temporal region, which gradually expanded and was occasionally scratched due to mild pruritus. Two years ago, there was no apparent trigger for the emergence of additional skin lesions; however, a number of dark-brown masses of varying sizes began to slowly grow. These lesions occasionally ulcerated and formed scabs independently, accompanied by itching, and remained untreated. The patient reported being in good health prior to this presentation and denied any family history of similar conditions.

**Figure 3:**
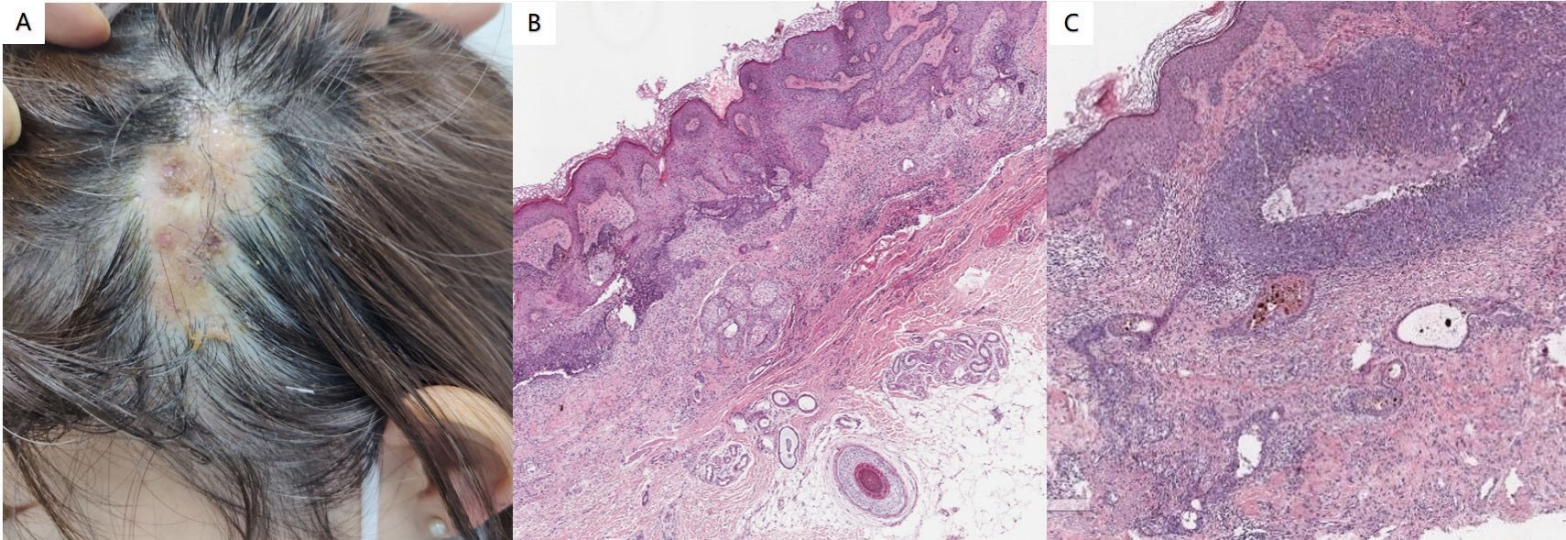
**(A)** Soft yellowish patches were observed in the left parieto-temporal region of the scalp. **(B)** Histopathological analysis revealed excessive epidermal keratosis, prolonged epidermal processes, and hyperplasia of sebaceous glands, which were present without corresponding hair follicles or sweat glands in the deep dermis and subcutaneous fat (hematoxylin and eosin staining; magnification:×100). **(C)** Clearly defined tumor masses were identified in the dermis, with some connected to the epidermis. These tumor masses consist of basal-like cells, and the surrounding cells are arranged in a palisade formation. Additionally, some tumor masses contain single or nested sebaceous differentiated cells interspersed within and around them (hematoxylin and eosin staining; magnification:×200).

Physical examination revealed no abnormalities within the system. Dermatological assessment identified a soft, yellowish patch measuring approximately 3.1 cm × 5.8 cm located on the left parietal and temporal regions of the scalp (**Figure 3A**). Histopathological analysis demonstrated excessive epidermal keratosis, prolonged epidermal processes, and hyperplasia of sebaceous glands without corresponding hair follicles, as well as sweat glands present in the deep dermis and subcutaneous fat (**Figure 3B**). Clearly defined tumor masses were observed in the dermis, some of which were connected to the epidermis. These tumor masses comprised basal-like cells, with surrounding cells arranged in a palisade formation. Additionally, some tumor masses exhibited a mixture of single or nested sebaceous differentiated cells both within and around them (**Figure 3C**). It is suitable for the diagnosis of NS complicated with BCC. This histopathological subtype of BCC is nodular.

The entire lesion was surgically excised. After confirming the clearance of the incisal margin and base, flap surgery was performed to close the wound, and there was no recurrence observed during the 1-year follow-up.

### Case 4

A 49-year-old man presented to a dermatology clinic with a left frontal plaque. The patient reported the presence of a large yellowish patch at the top of the left frontal area shortly after birth, accompanied by occasional pruritus. Over the past two years, several gray-black patches have developed at the base of the yellowish patch, with these lesions growing slowly and remaining asymptomatic, without itching or pain. The lesion has not been treated since its emergence. None of the skin lesions exhibited ulceration, bleeding, discharge of pus, or oozing fluid. The patient reported being in good health, with no significant personal or family medical history.

**Figure 4:**
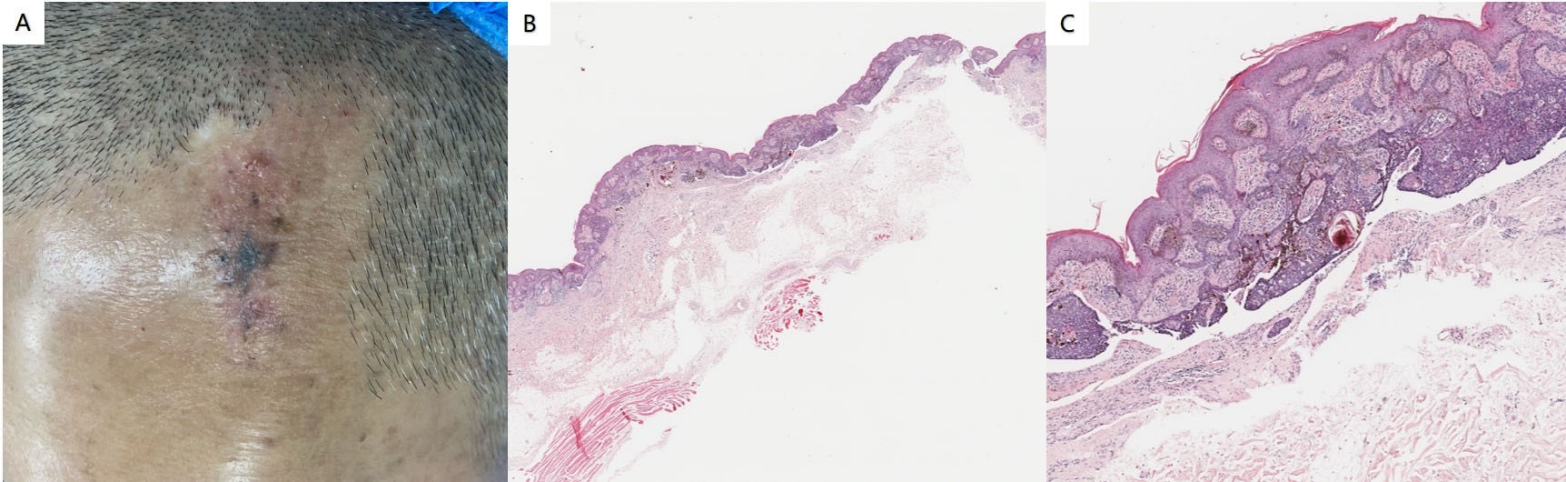
**(A)** A leathery patch is observed at the top of the left frontal region, characterized by a rough surface and medium quality. Additionally, several gray-black patches of varying sizes and smooth surfaces are present. **(B, C)** Histopathological findings revealed an epithelial tumor composed of basal-like cells located in the superficial layer of the dermis. This tumor exhibited pigmentation and interstitial fibrous tissue hyperplasia, with local tumors associated with epidermal hyperplasia. Furthermore, artificial contraction spaces were noted (hematoxylin and eosin staining; magnification: B×40, C×100).

Physical examination revealed no abnormalities in the system. Dermatological assessment identified a leathery patch measuring approximately 4.8 cm × 2.1 cm at the top of the left frontal area, characterized by a rough surface and medium quality. In addition to this patch, several gray-black lesions of varying sizes and smooth surfaces were observed, displaying slight elevation, uneven coloration, medium quality, and poor mobility (**Figure 4A**). Histopathological analysis indicated the presence of epithelial tumors composed of basal-like cells in the superficial layer of the dermis, accompanied by pigmentation and interstitial fibrous tissue hyperplasia. Local tumors were associated with epidermal hyperplasia, and artificial contraction spaces were evident (**Figures 4B, C**). The findings are consistent with a diagnosis of NS complicated by BCC. This histopathological subtype of BCC is a superficial phenotype.

The entire lesion was surgically excised, and a frozen section was obtained during the procedure. Following confirmation of clean margins at the incision site and base, a flap was utilized to close the wound. Post-operative recovery was uneventful, and after 6 months of follow-up, there was no evidence of recurrence.

## Discussion

NS is a benign congenital skin lesion characterized by clinically hairless, yellow-orange patches of varying sizes and shapes, most commonly located in sebaceous-rich areas such as the scalp and neck. Structurally, NS is classified as a hamartoma composed of epidermis, sebaceous glands, and apocrine glands, providing conditions conducive to the development of secondary tumors (4). A meta-analysis revealed that malignant tumors developed in 8% of 4,923 sebaceous nevi, while benign tumors were observed in 16% of the same cases (5). BCC, one of the most prevalent cutaneous malignancies, was identified in approximately 1.1% of 707 patients with secondary neoplasms associated with NS. Notably, NS located on the scalp accounted for 62.5% of these cases, whereas those on the face comprised 24.5% (2). The abnormal expression of various genes plays a significant role in the occurrence and progression of NS. Groesser et al. reported that 95% of patients exhibited mutations in the HRAS gene, and 5% had mutations in the KRAS gene among 65 sebaceous nevi, suggesting that NS is caused by postzygotic HRAS and KRAS mutations. Furthermore, somatic HRAS mutations have also been detected in various malignant tumors, indicating the oncogenic potential of this mutation. Activation of HRAS leads to the stimulation of the mitogen-activated protein kinase (MAPK) and phosphoinositide 3-kinase (PIK3)-Akt signaling pathways, resulting in increased cellular proliferation (6). Cornejo et al. also evaluated the clinicopathologic and molecular features of both Syringocystadenocarcinoma papilliferum and NS. RAS-activating mutations were detected in 50% of patients in lesion and HRAS mutations were also noted in the background lesion adjacent areas, emphasizing RAS mutations were associated with early oncogenic events and malignant transformation (7). Gambini et al. found that the characteristics of early-onset BCC may be caused by germline mutations in PTCH1 (8).

BCC typically manifests as small, pink or pearly white bumps that are translucent or waxy. It occurs more frequently in men than in women, particularly in individuals over the age of 50 (9). The primary risk factors for the development of BCC include ultraviolet radiation exposure, skin type, male sex, advanced age, long-term immunosuppression, and genetic mutations (10). Both RAS oncogene and patched gene mutations have been correlated with malignancies arising in NS (11). Loss of the patched 1 gene (PTCH1) and other mutations, including those in DNA damage repair genes and members of the PI3K-Akt pathway, have been implicated in BCC development. The loss of PTCH1 can deregulate the Hedgehog (HH) signaling pathway, leading to Smoothened (SMO) derepression and the activation of GLI transcription factors (12). Furthermore, tumorigenesis induced by the HH signaling pathway is enhanced by concomitant activation of the PI3K-Akt signaling pathway (13), including HRAS-activated PI3K-Akt signaling in NS. Changes in apoptosis pathways, such as increased expression of MAPK, contribute to the oncogenesis of BCC (14). Activation of RAS subfamily, including HRAS and KRAS, initiated a multi-step phosphorylation cascade that caused the activation of RAF/MEK/ERK1/2 signaling pathway, which subsequently translocated to the nucleus and regulated transcriptional programs associated with cell proliferation (15). Activation of RAS in HH-responsive cell lines resistance to inhibitors of the HH signaling pathway. Gene set enrichment analysis of resistant BCC exhibiting a diminished HH pathway signature showed increased RAS/MAPK pathway activation (16).Collectively, the evidence suggests that the MAPK and PI3K-Akt signaling pathways regulated in NS could influence the signaling pathways involved in BCC.

In addition to genetic mutations and the activation of signaling pathways, exposure to ultraviolet radiation and advanced age are the primary risk factors associated with BCC (17). We report four adult patients, all approximately 50 years old, with BCC arising in NS on the left side of the scalp. Areas exposed to ultraviolet radiation, such as the head and neck, are common sites for BCC development (18). Although the presence of hair on a healthy scalp can reduce ultraviolet radiation exposure from sunlight, the hairless nature of NS in our patients fails to effectively block ultraviolet radiation, leading to an increased incidence of BCC. Both ultraviolet A and B wavelengths, which cause DNA damage due to excessive ultraviolet radiation absorption by nuclear DNA, are now recognized as Class 1 carcinogens in humans and are associated with the extent of DNA damage (19). Under ultraviolet radiation conditions, keratinocytes may secrete various mediators, such as MAPK, which regulate gene expression (20) and contribute to the development of BCC. From infancy to puberty, the lesions of NS transition from yellow plaques to verrucous formations. In the third stage, the emergence of secondary tumors arising in NS typically occurs in late adulthood, which aligns with the ages of our four adult patients.

We reviewed the literature on the growth of various malignancies, including BCC arising in NS, over the last ten years. There is substantial evidence that NS may develop into tumors of various epidermal and adnexal origins. It is quite rare for more than two tumors to occur simultaneously in NS (**Table 1**), with most cases arising in sun-exposed areas such as the head and face. The majority of reported patients are middle-aged and elderly. The primary treatment for this condition is surgical resection.

**Table 1:** Summary of various malignancies including BCC arising in NS in the last ten years.

Reference	Diagnosis	Location	Age	Gender	Treatment method
Maty et al., 2020 (11)	BCC	Forehead	41	Male	Surgical excision
Kneiber et al., 2019 (21)	BCC	Face	32	Female	Surgical excision
Luo et al. 2024 (22)	BCC	Scalp	55	Male	Photodynamic therapy in combination with surgical excision
Watson et al., 2019 (23)	BCC	Trunk	82	Female	Local excisions, topical imiquimod, and close monitoring
David et al., 2021 (24)	BCC and sebaceoma	Scalp	65	Female	Surgical excision
Gupta et al., 2015 (25)	BCC and syringocystadenoma papilliferum	Face	27	Male	Surgical excision
Bostanci et al., 2020 (26)	BCC and sebaceous carcinoma	Left cheek	67	Female	Surgical excision
Hihara et al., 2022 (27)	BCC, sebaceous carcinoma, and apocrine adenocarcinoma	Scalp and neck	57	Male	Wide extended excision
Jiang et al. 2022 (28)	BCC and syringocystadenoma papilliferum	Scalp	46	Male	Surgical excision
Paninson et al. 2019 (29)	BBC	Scalp	38	Male	Surgical excision
Ferraz et al. 2024 (30)	BBC	Right forehead	41	Male	/
Nazzaro et al. 2015 (31)	BBC, tricholemmoma, sebaceous adenoma, and syringocystadenoma papilliferum	Scalp	41	Female	Surgical excision

BCC, basal cell carcinoma; NS, nevus sebaceous.

In the four cases reported in this paper, the disease was primarily located in the left cephalotemporal region of the patients, which seems to be consistent with the results reported in previous literature. Earlier studies indicated that BCC predominantly occurs in the head and face, with fewer cases observed in the trunk. Our findings suggest the need for further research focused on the cephalotemporal region, particularly the left side, where BCC appears to be more prevalent. The primary causative factor for BCC in the head and face is ultraviolet radiation (10, 17); however, the potential influence of other factors warrants additional investigation. The four patients discussed in this paper are middle-aged and elderly, aligning with previously reported cases. Furthermore, the observed gender differences among BCC patients indicate a need for more comprehensive research. The cases presented herein predominantly underwent surgical treatment, consistent with findings from earlier studies.

Pathologically, BCC subtypes are primarily categorized into nodular, adenoid, cystic, superficial, scleroderma-like, and pigmented types. Among these, the nodular type is the most prevalent, with the other subtypes being relatively rare. Based on clinical manifestations and pathological data, the four case subtypes discussed in this paper can be classified into nodular and superficial phenotypes. Specifically, cases 1, 2, and 3 exhibited the nodular type, while case 4 presented with the superficial phenotype. The possible reason is that nodular BCC typically has a larger volume and more severe clinical manifestations, which often draw patients’ attention and prompt them to seek medical treatment promptly. Additionally, due to the large number of patients with this type, clinicians have extensive experience in its diagnosis and treatment, leading to a high diagnostic accuracy rate for this subtype.

Although several treatments for NS exist, including curettage, cauterization, cryotherapy, and photodynamic therapy, surgical excision remains the most common method to ensure complete eradication (29). The etiology of NS complicated by BCC is not well understood; however, it is speculated that local malignant lesions may arise from improper treatment of sebaceous nevus in the early stages, repeated cryotherapy, local irritation, and infection. Consequently, it is imperative for dermatologists to apply appropriate early treatment for NS to prevent the development of malignant lesions resulting from inadequate management.

All 4 cases in this paper were treated with Mohs micrographic surgery, which is a more complete examination of the entire surgical margin of excised skin malignancies than traditional surgical methods (32). Mohs micrographic surgery has the advantage of being cost-effective, not only with smaller excisions and better aesthetic and functional results, but also with a lower risk of recurrence compared to traditional surgery (33). In addition, all patients in this study were repaired using *in situ* flap reconstruction, a commonly used surgical technique for repairing tissue defects caused by trauma, tumor resection, or other reasons. This surgical method is advantageous for preserving local blood supply, and it is characterized by simplicity, rapid recovery, and high aesthetic value. Postoperative complications and follow-up are critical components in ensuring the success of the surgery and the recovery of patients. Common postoperative complications include flap necrosis, infection, hematoma, fluid accumulation beneath the flap, edge necrosis, sensory disturbances, pigmentation changes or loss, and scar hyperplasia. The purpose of postoperative follow-up is to monitor flap survival, promptly detect and address complications, and evaluate the surgical outcome. Early follow-up (1-2 weeks post-surgery) involves observing the color, temperature, and capillary refill of the flap, as well as checking for signs of hematoma, infection, or other complications. Patients should be guided in proper wound care and functional exercises. Intermediate follow-up (weeks to months post-surgery) focuses on assessing flap survival, sensory recovery, and functional outcomes. Additionally, the formation of scars should be monitored, and scar treatment should be administered as necessary. Late follow-up (months to years post-surgery) entails ongoing monitoring of the flap’s long-term effects and patient satisfaction, along with checks for tumor recurrence or other long-term complications. Based on the patient’s needs, any necessary repair or cosmetic procedures can be performed. The specific frequency and content of follow-up should be tailored to the individual patient’s circumstances and the type of surgery performed. In this article, four patients underwent flap reconstruction surgery, and their wounds healed well. Throughout the follow-up period, there were no significant signs of necrosis, infection, hematoma, or sensory disturbances, and the status of flap survival was satisfactory. Follow-up efforts primarily focused on the formation and repair of scar at the surgical site.

Watson’s team reported a patient with five BCCs located within a large NS on the trunk, who was treated with local excision, topical imiquimod, and close monitoring. Furthermore, regular follow-up was deemed necessary for patients with NS (23). In addition to excision, photodynamic therapy was proposed as a minimally invasive option for malignant tumors. Luo et al. presented a case of BCC arising within an NS that was successfully treated with photodynamic therapy following complete excision of the plaque (22). The patient demonstrated significant resolution of the lesions within six months, indicating high efficacy and favorable aesthetic outcomes. There have also been increasing reports regarding the surgical excision of various malignant tumors in NS. Jiang and colleagues reported a case of syringocystadenoma papilliferum and BCC arising in NS. The initial diagnosis was proposed based on dermoscopic and reflectance confocal microscopic findings, followed by total excision of the lesion (28). Bostanci et al. reported a case of excision of sebaceous carcinoma and BCC arising in NS, based on dermoscopic and histopathological examination (26). Additionally, sebaceoma and BCC within NS have also been documented (24). Although most secondary neoplasms occur after puberty, Altaykan et al. reported a case of BCC arising in NS on the scalp of a 10-year-old boy, suggesting that preventive treatment for NS is necessary (34).

Early prophylactic removal of NS is currently recognized as a treatment option; however, the timing of this intervention remains controversial. Given that NS predominantly occurs on the head and face, some scholars argue that excision during childhood can concurrently reduce the formation of keloids in patients, thereby minimizing aesthetic damage. Conversely, other scholars contend that NS rarely undergoes malignant transformation in childhood. If surgical resection is performed at this time, the use of general anesthesia and the complexity of the surgical process may pose certain risks to pediatric patients. Therefore, some experts advocate delaying preventive resection until adolescence (3). Furthermore, complications following surgical excision of NS are more likely to occur in children than in adults, suggesting that excision could be postponed until after childhood (35). Overall, the malignant transformation of NS mainly occurs in adulthood, so we recommend that preventive resection of NS should be performed before this. However, as the risk of malignant transformation increases over time, it is strongly recommended to undergo excision during adolescence (3).

## Data Availability

The datasets presented in this article are not readily available because the datasets presented in this article are not readily available because of ethical and privacy restrictions. Requests to access the datasets should be directed to YN, niyao899@163.com.
